# Azithromycin and its non-antibacterial derivative EP317 attenuate select aspects of ventilator-induced lung injury in rats

**DOI:** 10.1371/journal.pone.0358745

**Published:** 2026-09-24

**Authors:** Sigurbergur Karason, Arni Asbjarnarson, Saevar Ingthorsson, Kolbrun Bjork Jonasdottir, Jennifer Kricker, Thorarinn Gudjonsson, Jon Petur Joelsson

**Affiliations:** 1 Department of Anaesthesia and Intensive Care, Landspitali-University Hospital, Reykjavik, Iceland; 2 Stem Cell Research Unit, BioMedical Centre, School of Health Sciences, University of Iceland, Reykjavik, Iceland; 3 Faculty of Nursing and Midwifery, University of Iceland, Reykjavik, Iceland; 4 EpiEndo Pharmaceuticals, Reykjavik, Iceland; 5 Department of Laboratory Hematology, Landspitali-University Hospital, Reykjavik, Iceland; University of Michigan, UNITED STATES OF AMERICA

## Abstract

Ventilator-induced lung injury (VILI) is a significant complication of mechanical ventilation, contributing to morbidity and mortality in critically ill patients. Excessive mechanical stress triggers an inflammatory cascade, leading to lung damage, pulmonary oedema, and systemic complications. While macrolide antibiotics such as azithromycin (AZM) have demonstrated anti-inflammatory and epithelial barrier strengthening effects, their use is limited by concerns regarding antibiotic resistance. EP317, an azithromycin-derived compound with negligible antibacterial activity, was designed to retain the immunomodulatory and epithelial barrier enhancing effects of AZM while mitigating the risk of bacterial resistance. This study investigated the effects of AZM and EP317 in a rat model of VILI. Forty-eight rats were divided into six groups (N = 8 per group), receiving either placebo, AZM, or EP317 (5 mg/kg daily for seven days), with half of the animals subjected to mechanical ventilation with peak airway pressure at 35 cmH_2_O. Both compounds attenuated ventilation-induced pulmonary oedema, reflected in a smaller increase in lung wet/dry ratio, whereas ventilation-induced increases in total protein in bronchoalveolar lavage fluid (BALF) were not significantly reduced by either treatment. Cytokine profiling in plasma (L-Selectin, PAI-1, RANTES, MCP-3, IL-6, SCF, IL-17a, RAGE, MIP-3α, IL-13, G-CSF, IL-10, M-CSF, IFN-γ, GRO-KC and MMP9) revealed that both treatments attenuated inflammatory responses, while cytokine levels in BALF in ventilated animals were either unchanged or increased. Survival rates were improved in the treatment groups. Histological analysis indicated increased oedema in ventilated animals, likely due to the high-pressure ventilation, while drug-treated groups exhibited reduced oedema. Global gene expression profiling by RNA sequencing revealed robust upregulation of inflammatory and stress-response pathways in ventilated animals, with partial attenuation following AZM or EP317 pre-treatment. These findings suggest that EP317 and AZM offer protective effects against VILI. EP317 presents a promising alternative to conventional macrolide therapy by circumventing antibiotic resistance concerns.

## Introduction

Mechanical ventilation is a life-saving intervention for patients with respiratory failure; however, it carries the risk of ventilator-induced lung injury (VILI) characterized by increased permeability of the alveolar-capillary barrier, inflammation, pulmonary oedema and worsening of lung function [1]. During ventilation, excessive mechanical strain contributes to baro- and volu-trauma, while the repeated opening and closing of alveoli causes atelectrauma of the lung tissue, all of which lead to the release of pro-inflammatory cytokines and further exacerbate lung injury. Leakage of these inflammatory factors into the systemic circulation can affect other organs and lead to multiorgan failure, a process known as biotrauma [1]. Despite advancements in ventilatory strategies aimed at minimizing harm, VILI remains a significant clinical challenge, underscoring the need for pharmacological interventions that can mitigate its effects.

In vitro studies have shown that macrolides enhance lung epithelial integrity through mechanisms like promoting tight junction protein and stabilizing cell membranes [2]. Animal studies have demonstrated that the anti-inflammatory and immunomodulatory effects of macrolides can reduce VILI [3,4] and effects on oxidative stress have also been proposed [5]. The macrolide azithromycin (AZM) has also been suggested to improve mortality and shorten ventilator time in patients with acute respiratory distress syndrome (ARDS) [6].

However, AZM is classified by the WHO AWaRe as a “Watch” category antibiotic, and its widespread use raises concerns about bacterial resistance, limiting its long-term clinical applicability [7]. To address this limitation, EP317 was developed as a macrolide-based derivative of AZM that retains its immunomodulatory effects while lacking antibacterial properties, thereby eliminating selective pressure for antibiotic resistance. Although EP317 has been explored as a potential epithelial barrier-promoting agent in ulcerative colitis [8], the potential of EP317 as a therapeutic agent in VILI has not been previously explored.

In this study, we investigated the effects of AZM and EP317 on VILI outcomes in a rat model. We employed a controlled experimental design using 48 rats divided into six groups, receiving either placebo, AZM, or EP317 (5 mg/kg daily for 7 days), with or without mechanical ventilation with airway peak pressure at 35 cmH_2_O. Our primary objectives were to evaluate inflammatory responses through cytokine profiling in plasma and bronchoalveolar lavage fluid (BALF), assess survival outcomes, quantify pulmonary oedema, and analyse histological changes associated with VILI. By elucidating the potential protective effects of these pharmacological treatments, our findings may contribute to the development of targeted therapies for VILI, offering a promising avenue for improving outcomes in ventilated patients while addressing concerns of antibiotic resistance.

## Materials and methods

### Ethics statement

All animal procedures were prospectively approved by the Icelandic Food and Veterinary Authority (MAST), approval number 2402316. No institutional IACUC was required as Iceland operates under national veterinary authority oversight. Anaesthesia was induced and maintained using ketamine (75 mg/kg) and xylazine (10 mg/kg) intraperitoneally, supplemented with isoflurane (2%) via nasal mask, while tracheotomy was performed. Euthanasia was performed by intraperitoneal injection of pentobarbital and lidocaine at the conclusion of the experimental protocol. All research staff held FELASA accreditation in laboratory animal science.

### Animals

Forty-eight male Sprague Dawley rats were purchased from Taconic Biosciences, Denmark, at ages of 8–10 weeks. Rats were acclimatized for 5 days and were housed in environmentally enriched cages in groups of four, under a light/dark regime of 12:12 hours with food and water ad libitum.

Animals were housed in environmentally enriched, group-housed cages (four per cage) under a 12:12 hour light/dark cycle with ad libitum food and water. Their health and behaviour (general appearance, activity, body weight, and signs of distress at the oral gavage site) were monitored daily by trained animal facility staff throughout the 5-day acclimatization period and the 7-day dosing period. Body temperature was monitored continuously via rectal thermometer during the ventilation procedure. No animals showed signs of ill health, weight loss, or distress prior to the ventilation protocol, and none required removal from the study before the planned endpoint. Animals were maintained under deep surgical anaesthesia (ketamine/xylazine plus 2% isoflurane) for the full duration of the ventilation procedure and were not permitted to recover before euthanasia; anaesthetic depth was confirmed by absence of pedal withdrawal reflex prior to tracheotomy, and supplemental anaesthetic doses were administered every 20–30 minutes as described in the Ventilation protocol below. The pre-defined humane endpoint (heart rate below 20 beats per minute by continuous ECG) ensured immediate euthanasia of any animal showing signs of imminent decompensation, minimizing suffering.

Because tissue collection for wet/dry ratio, RNA sequencing, and histology is incompatible with terminal BALF and plasma sampling from the same animal, each of the six groups (N = 8) was divided into two sub-cohorts of four rats: one sub-cohort was used for terminal BALF and tail vein plasma collection, and the other for lung lobe collection (left lung for RNA sequencing, right middle lobe for wet/dry ratio, right inferior lobe for histology), as indicated in Table 1. Ventilation was performed using four small-rodent mechanical ventilators (Harvard Apparatus); given this throughput limit, the 48 animals were ventilated across multiple sequential sessions rather than simultaneously.

**Table 1:** Rat groupings.

Group	Abbreviation	Treatment	Ventilation	N
1	PC	Placebo (citrate buffer)	Non-ventilated	8
2	PV	Placebo (citrate buffer)	35 cmH_2_O PIP	8
3	AC	AZM (5 mg/kg/day × 7 days)	Non-ventilated	8
4	AV	AZM (5 mg/kg/day × 7 days)	35 cmH_2_O PIP	8
5	EC	EP317 (5 mg/kg/day × 7 days)	Non-ventilated	8
6	EV	EP317 (5 mg/kg/day × 7 days)	35 cmH_2_O PIP	8

### Dosing regime

In this study, adult Sprague-Dawley rats (weighing 250–300 g) were administered AZM or EP317. Drug treatments were administered via oral gavage (5 mg/kg per day for 7 consecutive days). Both compounds were dissolved in citrate buffer, which was also used as the placebo control. The human equivalent dose, calculated by allometric scaling, corresponds to just under 1 mg/kg, representing a modest treatment regimen.

The dosing regimen of 5 mg/kg/day for 7 days was selected based on previous pharmacokinetic studies demonstrating that AZM exhibits extensive tissue distribution and a prolonged elimination half-life in rats [9]. Specifically, tissue concentrations of AZM have been shown to increase proportionally with the administered dose, with higher concentrations achieved following repeated dosing compared with single-dose administration. The dosing strategy in this study aimed to achieve therapeutic tissue concentrations while minimizing potential adverse effects associated with high single doses [10].

EP317, a novel derivative of AZM lacking antibacterial activity but retaining its non-antibacterial properties, was administered using the same dosing regimen as AZM. This approach facilitated a direct comparison of their effects on VILI outcomes. EP317 was designed and manufactured by EpiEndo Pharmaceuticals.

Animals were assigned to treatment groups by simple randomization at the time of cage allocation. Investigators performing biochemical assays (Luminex multiplex cytokine analysis, BCA total protein assay) and RNA sequencing data analysis were not blinded to group assignment, as these are objective, instrument-based quantitative measurements performed against fixed standard curves and analysis pipelines, with no scope for subjective operator judgement to influence the recorded value. Histopathological assessment, which requires a degree of subjective interpretation and is therefore most susceptible to observer bias, was performed by an independent, blinded pathologist at PatConsult (patconsultlab.com), as stated in the Histology section below.

### Ventilation protocol

Rats were anaesthetised by administering ketamine (75 mg/kg) and xylazine (10 mg/kg) intraperitoneally (i.p.), supplemented by inhalation of isoflurane (2%) via nasal mask, while tracheotomy was performed. Rats were further supplemented every 20–30 minutes with one-third of the initial dose of ketamine and xylazine i.p. After anaesthesia was confirmed, a tracheotomy was performed and a cannula (#73–2855 Harvard Apparatus, OD 2.5 mm, L 45 mm) was inserted, then rats were connected to small rodent mechanical ventilators (Harvard Apparatus) and ventilated with an injurious ventilator setting: peak inspiratory pressure (PIP) of 35 cmH_2_O, positive end-expiratory pressure (PEEP) at 5 cmH_2_O, 60 breaths per minute, 50:50 inspiration/expiration ratio and 21% oxygen in inspired air. In this manner a stable airway pressure was maintained even though compliance of the lungs would change. The ventilation parameters were selected based on a systematic review of rat VILI models, which identified this pressure range as appropriate for inducing reproducible lung injury [11]. Animals were positioned supine on heated mats with body temperature monitored via a rectal thermometer. Animals were ventilated for 2 hours or until death. Death was determined via electrocardiogram (ECG) monitoring (Iworx, IX-BIO8). The pre-defined humane endpoint was a heart rate below 20 beats per minute as measured by continuous ECG. Upon reaching this criterion, the animal was immediately removed from the ventilator and euthanized by intraperitoneal injection of pentobarbital and lidocaine within one minute. No animals died before meeting the humane endpoint criterion.

Non-ventilated rats underwent the same procedure, receiving a tracheotomy, but were not connected to a ventilator and were euthanized immediately after blood samples were collected with an i.p. injection of pentobarbital and lidocaine. Ventilated rats that did not reach the humane endpoint were euthanized in the same manner at the end of the 2-hour ventilation period. In total, 48 animals were used; 7 ventilated animals (6 placebo-treated, 1 AZM-treated) were euthanized upon reaching the humane endpoint during the protocol, and the remaining 41 animals were euthanized at the conclusion of the experiment. Rats were split into 6 groups of 8: three non-ventilated groups (Placebo, AZM or EP317) and three ventilated groups (Placebo, AZM or EP317), as shown in Table 1. Groups were designated the following abbreviations: PC = Placebo – non-ventilated, PV = Placebo – ventilated, AC = AZM pre-treated – non-ventilated, AV = AZM pre-treated – ventilated, EC = EP317 pre-treated – non-ventilated and EV = EP317 pre-treated – ventilated. Four rats from each group were used for collection of BALF and tail vein blood and four rats were used for lung lobe collections.

### Wet/dry ratio

Right middle lobes were removed from the rats and weighed, then immediately placed in an incubator at 80°C for 72 hours for drying, the lobes were then re-weighed and the ratio of wet/dry was calculated.

### Cytokine analysis

Cytokine analyses were performed on both BALF and plasma samples. BALF was collected via the tracheal cannula: three injections of 3 mL saline each were administered and collected from the lungs, after which the samples were aliquoted and stored at −80°C. Blood was collected via the tail vein 90 minutes after ventilation had started or before BALF collection in the non-ventilated rats. Blood was collected into EDTA tubes, centrifuged at 10,000 rpm for 5 minutes and the resulting plasma was collected and stored at 4°C.

Samples were assayed using Luminex xMAP Technology. Assays were multiplexed into analyte-specific color-coded microspheres to attain distinct fluorescent signatures for each assay combination. Assay preparation and evaluation were performed following standard operating procedures for bead-based sandwich ELISA. Samples were brought to room temperature immediately before the performance of the assay. BALF samples were diluted 1:2 using sample diluent, except for the RAGE assay whereby the BALF samples were diluted 1:16 prior to measurement. BALF samples were assayed in duplicate. Plasma samples were diluted 1:4 using sample diluent and analysed in single measurements. A volume of 35 μL per sample/standard/blank/control was used in the assay.

### Total protein concentration

Total protein concentration in BALF was measured using the Pierce Dilution-Free Rapid Gold BCA Protein Assay kit (cat# A55860), according to the manufacturer’s protocol. In short, 200 μL of Rapid Gold BCA Reagent working reagent was mixed with 10 μL of BALF sample or protein standard and the absorbance was measured at 480 nm using a plate reader.

### RNA sequencing

The left lung was removed from the rats and placed immediately in Trizol. Samples were stored at −80°C and sent for RNA sequencing using DNBSEQ Eukaryotic Strand-Specific Transcriptome Resequencing (BGI Tech Solutions, Hong Kong). Sequencing was performed using paired-end 150 base pair (PE150) reads. Quantification of RNA transcript expression was carried out using Kallisto (v0.46.1), and Sleuth v0.30 was utilized for gene expression estimation computing. Pre-ranked gene lists were the prepared by expression difference significance. Gene set enrichment analysis (GSEA) was performed using Hallmark gene sets (GSEA, v4.3.2) [12,13].

### Histology

The right inferior lobe was collected from the rats and stored in formalin. Lungs were processed for histological analysis by embedding in paraffin wax before cross-sectioning using a microtome. Histopathological assessment of H&E-stained samples was performed by PatConsult (patconsultlab.com) in a blinded manner.

### Statistical analysis

Data were analysed using one-way ANOVA with Tukey’s post-hoc test across all six groups. This approach was selected to enable direct pairwise comparisons between all groups, with the key comparisons of interest being between ventilated and non-ventilated animals within each treatment group (to assess the effect of ventilation), and between the three treatment groups within the ventilated condition (to assess the protective effect of each compound). Statistical significance was determined using GraphPad Prism 10.6.1. Error bars represent the standard deviation of the means. Exact p-values are reported where available; asterisk notation (*p < 0.05, **p < 0.01, ***p < 0.001, ****p < 0.0001) is used in figures. Calculations for the statistical significance for the differential gene expression were calculated using the Wald test in Sleuth. All data are presented as mean ± SD unless otherwise stated.

Sample size (n = 8 animals per group) was determined based on effect sizes reported in prior published rat models of ventilator-induced lung injury, as summarized in our systematic review of the field [11], which identified n = 6–10 animals per group as sufficient to detect moderate-to-large effects on survival, oedema, and inflammatory endpoints in similar VILI models.

**Fig 1:**
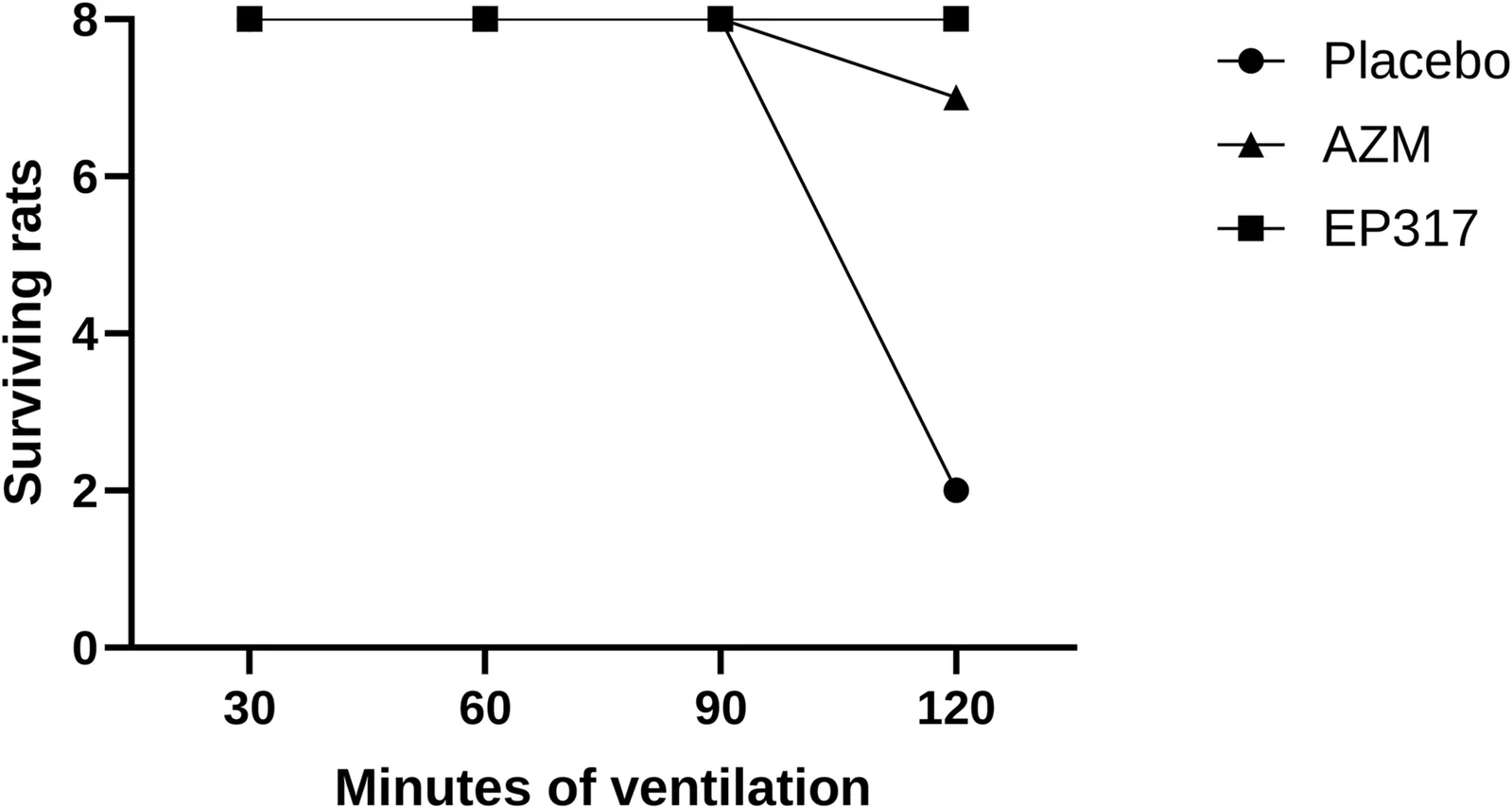
EP317 pre-treatment improves survival in ventilated rats subjected to injurious mechanical ventilation. Survival rates of rats ventilated with peak inspiratory pressure (PIP) of 35 cmH_2_O and positive end-expiratory pressure (PEEP) of 5 cmH_2_O over a 120-minute observation period. Animals were pre-treated with placebo (n = 8), azithromycin (AZM, n = 8), or EP317 (n = 8) prior to mechanical ventilation. All animals survived to 90 minutes. Between 90 and 120 minutes, 6/8 placebo-treated rats died, 1/8 AZM-treated rats died, and 8/8 EP317-treated rats survived. EP317 provided complete protection against ventilation-induced mortality during the observation period.

Survival (Fig 1) was assessed descriptively as the number and proportion of animals surviving within each treatment group at 30-minute observation checkpoints. Because animal status was recorded at these checkpoints even though Electrocardiogram was used for continuous monitoring, and given the small group size (n = 8 per group), no formal statistical test was applied to the survival data; group differences are reported as raw survival counts and proportions only.

Because ventilation status and treatment are both experimental variables in a factorial design, all quantitative endpoints (lung wet/dry ratio, BALF total protein, and all BALF and plasma cytokines) were additionally re-analysed using two-way ANOVA (Treatment × Ventilation) with Type II sums of squares, to formally test the main effects of treatment and ventilation and their interaction. Two-way ANOVA results are reported in the Results text alongside the corresponding one-way comparisons. The one-way ANOVA with Tukey’s post-hoc test described above is retained in the figures for the pre-specified pairwise comparisons of primary interest (ventilated versus non-ventilated within each treatment; treatment-versus-treatment within the ventilated condition).

## Results

### Treatment with either AZM or EP317 resulted in improved survival and reduced pulmonary oedema for ventilated rats

Survival rates of ventilated rats demonstrated substantial differences between treatment groups over the 2-hour observation period. All rats survived 90 minutes of ventilation but after that 6 of the 8 rats treated with placebo died within the next 30 minutes and 1 treated with AZM, while all rats treated with EP317 survived the 120 minutes (Fig 1).

**Fig 2:**
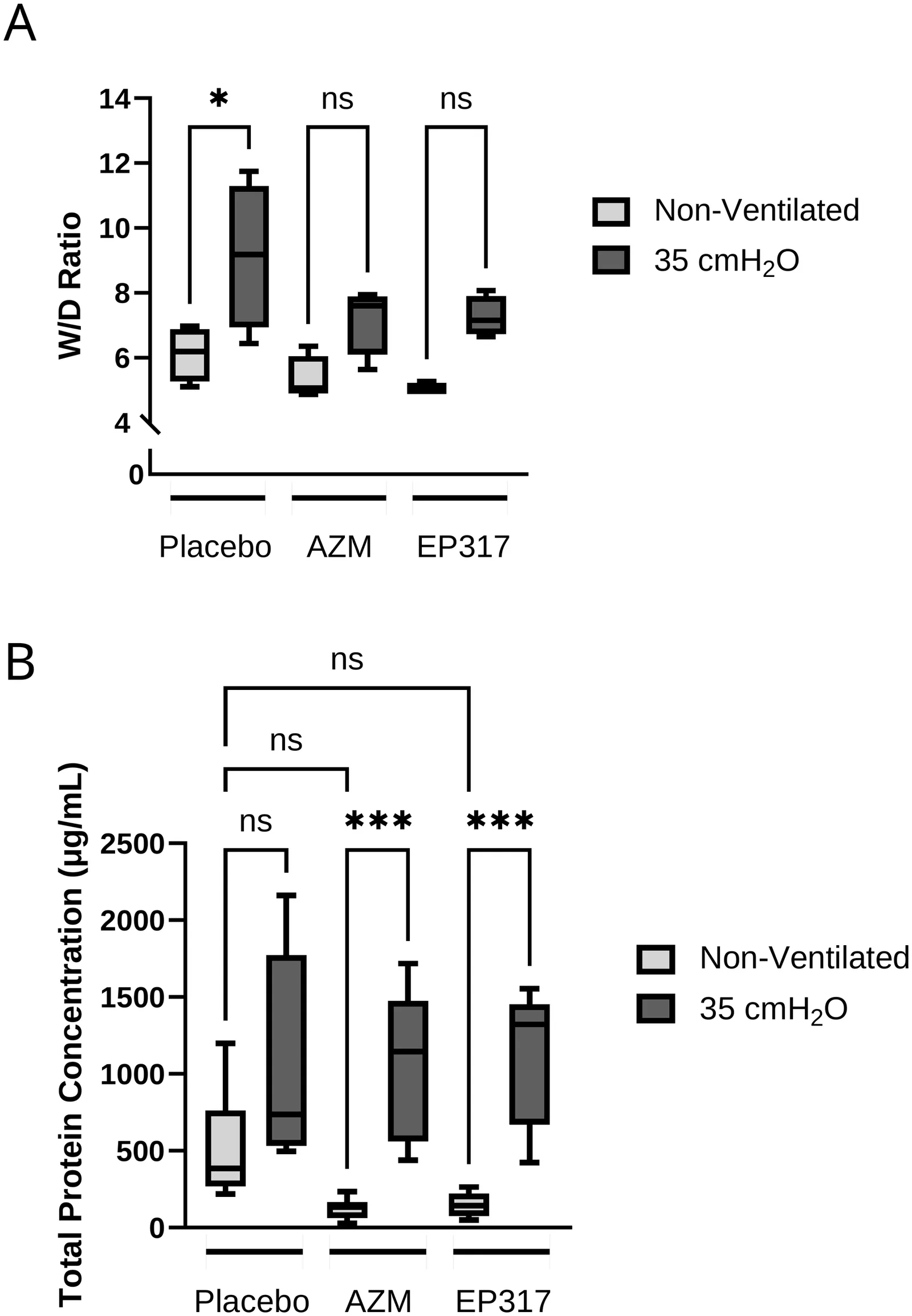
Pre-treatment with AZM or EP317 prevents ventilation-induced pulmonary oedema and modulates alveolar-capillary barrier function. (a) Lung wet-to-dry (W/D) weight ratios measured immediately after sacrifice. Mechanical ventilation significantly increased W/D ratio in placebo-treated animals (PV) compared to non-ventilated controls (PC) (p < 0.05), indicating fluid accumulation. No significant increase in W/D ratio was observed in AZM-ventilated (AV) or EP317-ventilated (EV) groups compared to their respective non-ventilated controls (AC and EC). (b) Total protein concentration in bronchoalveolar lavage fluid (BALF) as a marker of alveolar-capillary barrier integrity. Ventilation significantly increased BALF protein in both AZM and EP317 groups compared to non-ventilated controls (p < 0.001 for both), while the placebo group showed a non-significant trend. Data are presented as mean ± SD. Statistical analysis was performed using one-way ANOVA with Tukey’s post hoc test. *p < 0.05, **p < 0.01, ***p < 0.001.

Pulmonary oedema and alveolar-capillary barrier disruption were assessed by measuring the lung wet-to-dry (W/D) weight ratio and total protein concentration in BALF, respectively (Fig 2a and 2b). In placebo-treated animals, mechanical ventilation with peak airway pressure at 35 cmH_2_O caused a significant increase in W/D ratio compared to non-ventilated controls (p < 0.05), indicating marked fluid accumulation in the lung tissue. In contrast, no significant differences in W/D ratio were observed between ventilated and non-ventilated animals in either the AZM or EP317 treatment groups. To further assess barrier integrity, total protein concentration in BALF was measured. While ventilation in the placebo group led to a visible increase in protein content, this trend did not reach statistical significance. However, ventilation in both AZM- and EP317-treated animals resulted in significantly increased BALF protein concentrations compared to their respective non-ventilated controls (p < 0.001 for both). Notably, the non-ventilated AZM and EP317 groups exhibited consistently lower protein levels than placebo controls, although these differences were not statistically significant. Two-way ANOVA confirmed that ventilation was the dominant driver of both increased wet/dry ratio and BALF total protein (p < 0.001 for both), with a significant but modest main effect of treatment on wet/dry ratio (p = 0.044) and no significant treatment effect on total protein (p = 0.606), and no significant treatment × ventilation interaction for either endpoint. This indicates that AZM and EP317 pre-treatment did not statistically block ventilation-induced macromolecular permeability (as measured by BALF total protein), but was associated with a smaller net increase in lung wet/dry ratio. We interpret this dissociation as evidence that pre-treatment may support active alveolar fluid clearance (net fluid balance, reflected in wet/dry ratio) to a greater extent than it seals paracellular protein leak (reflected in BALF total protein) — two mechanistically distinct components of alveolar-capillary barrier function (see Discussion).

### Increased expression of inflammatory mediators affected by mechanical ventilation are systemically ameliorated by treatments with AZM or EP317

#### Cytokine response in BALF.

**Fig 3:**
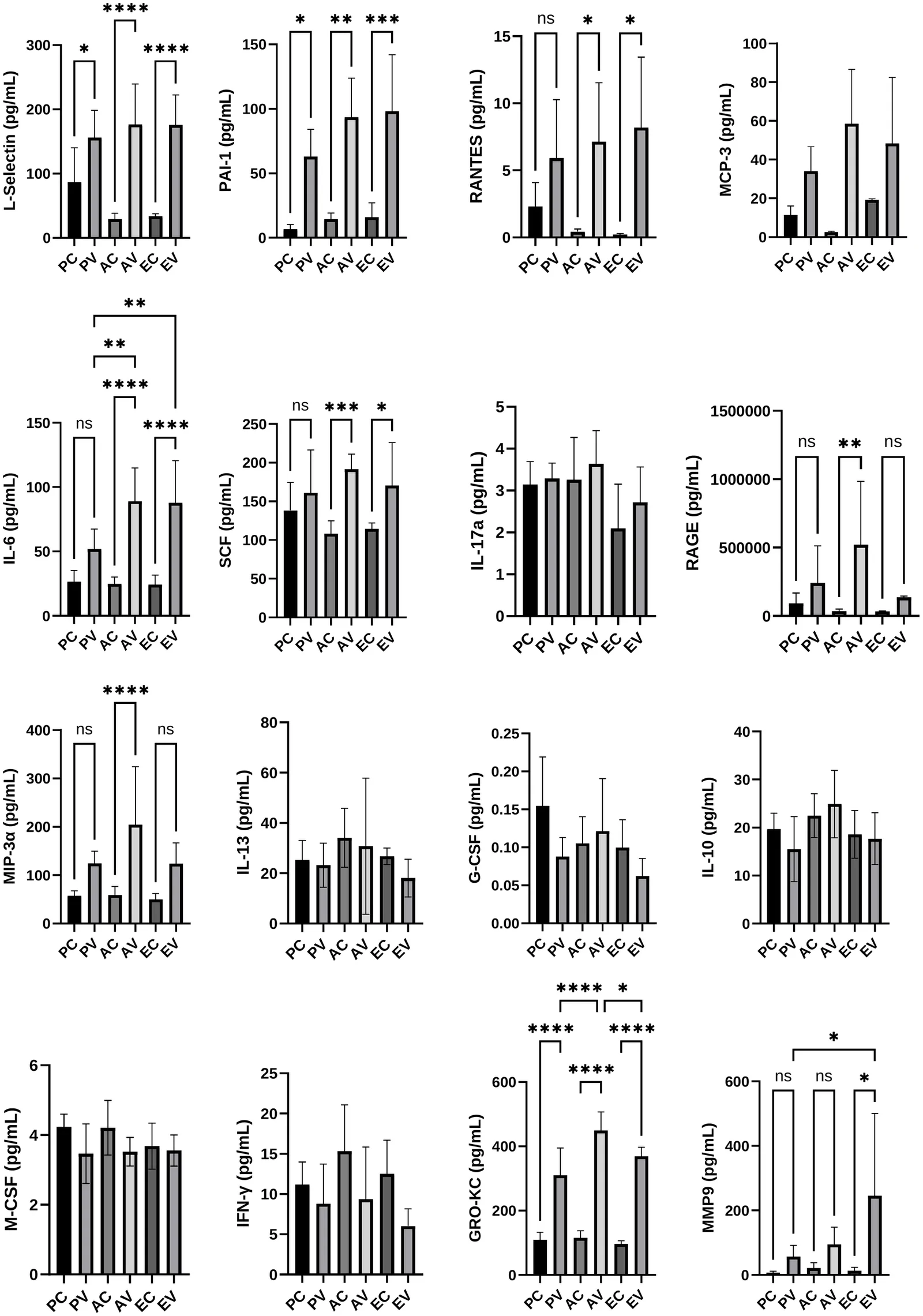
Mechanical ventilation induces a robust pro-inflammatory cytokine response in BALF that is not significantly attenuated by AZM or EP317 pre-treatment. Multiplex analysis of cytokine and chemokine concentrations in BALF collected at the end of the 120-minute ventilation period. Placebo-ventilated animals (PV) showed significant elevations in L-Selectin, plasminogen activator inhibitor-1 (PAI-1), RANTES, interleukin-6 (IL-6), stem cell factor (SCF), receptor for advanced glycation end products (RAGE), macrophage inflammatory protein-3α (MIP-3α), growth-regulated oncogene/keratinocyte chemoattractant (GRO-KC), and matrix metalloproteinase-9 (MMP9) compared to non-ventilated placebo controls (PC). Pre-treatment with AZM (AV) or EP317 (EV) did not significantly reduce these ventilation-induced cytokine elevations in BALF. Data are presented as mean ± SD. Statistical analysis was performed using one-way ANOVA with Tukey’s post hoc test. *p < 0.05, **p < 0.01, ***p < 0.001, ****p < 0.0001.

Mechanical ventilation caused a pronounced increase in pro-inflammatory and injury-associated cytokines in the BALF of placebo-treated animals (PV) (Fig 3). Notably, levels of L-Selectin, PAI-1, RANTES, IL-6, SCF, RAGE, MIP-3α, GRO-KC, and MMP9 were significantly elevated in ventilated animals compared to their non-ventilated counterparts (PC), consistent with acute pulmonary inflammation and epithelial injury. Pre-treatment with either AZM or EP317 did not attenuate these elevations to a significant degree.

#### Systemic cytokine response in plasma.

**Fig 4:**
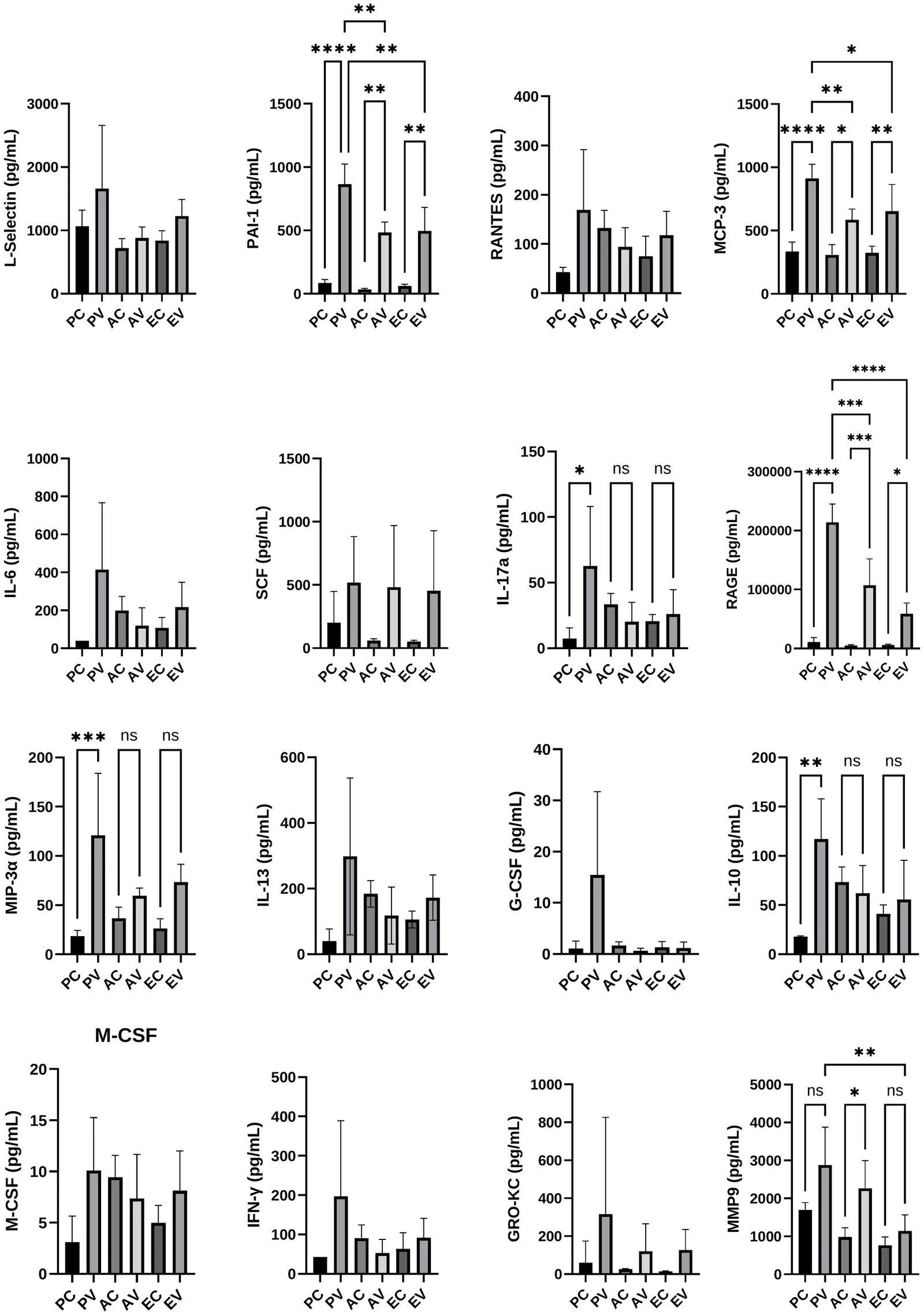
AZM and EP317 pre-treatment significantly attenuate systemic inflammatory responses to mechanical ventilation. Multiplex analysis of plasma cytokine and chemokine concentrations measured at the end of the experimental protocol. Mechanical ventilation in placebo-treated rats (PV) resulted in significant systemic increases in PAI-1, monocyte chemoattractant protein-3 (MCP-3), interleukin-17a (IL-17a), RAGE, MIP-3α, and interleukin-10 (IL-10) compared to non-ventilated controls (PC), with similar trends observed for L-Selectin, RANTES, IL-6, IL-13, granulocyte colony-stimulating factor (G-CSF), interferon-γ (IFN-γ), GRO-KC, and MMP9. Pre-treatment with either AZM (AV) or EP317 (EV) significantly attenuated the ventilation-induced increases in PAI-1, MCP-3, IL-17a, RAGE, MIP-3α, and IL-10, with consistent trends across additional cytokines. Data are presented as mean ± SD. Statistical analysis was performed using one-way ANOVA with Tukey’s post hoc test. *p < 0.05, **p < 0.01, ***p < 0.001, ****p < 0.0001.

In plasma (Fig 4), ventilation of placebo-treated rats (PV) led to significant increases in PAI-1, MCP-3, IL-17a, RAGE, MIP-3α and IL-10, with similar clear trends seen in L-Selectin, RANTES, IL-6, IL-13, G-CSF, IFN-γ, GRO-KC and MMP9. Importantly, pre-treatment with AZM or EP317 (AV/EV) significantly attenuated the ventilation-induced increases in plasma levels of PAI-1, MCP-3, IL-17a, RAGE, MIP-3α and IL-10, with similar trends observed across the other cytokines.

### Pre-treatment with AZM or EP317 results in less alveolar oedema

**Fig 5:**
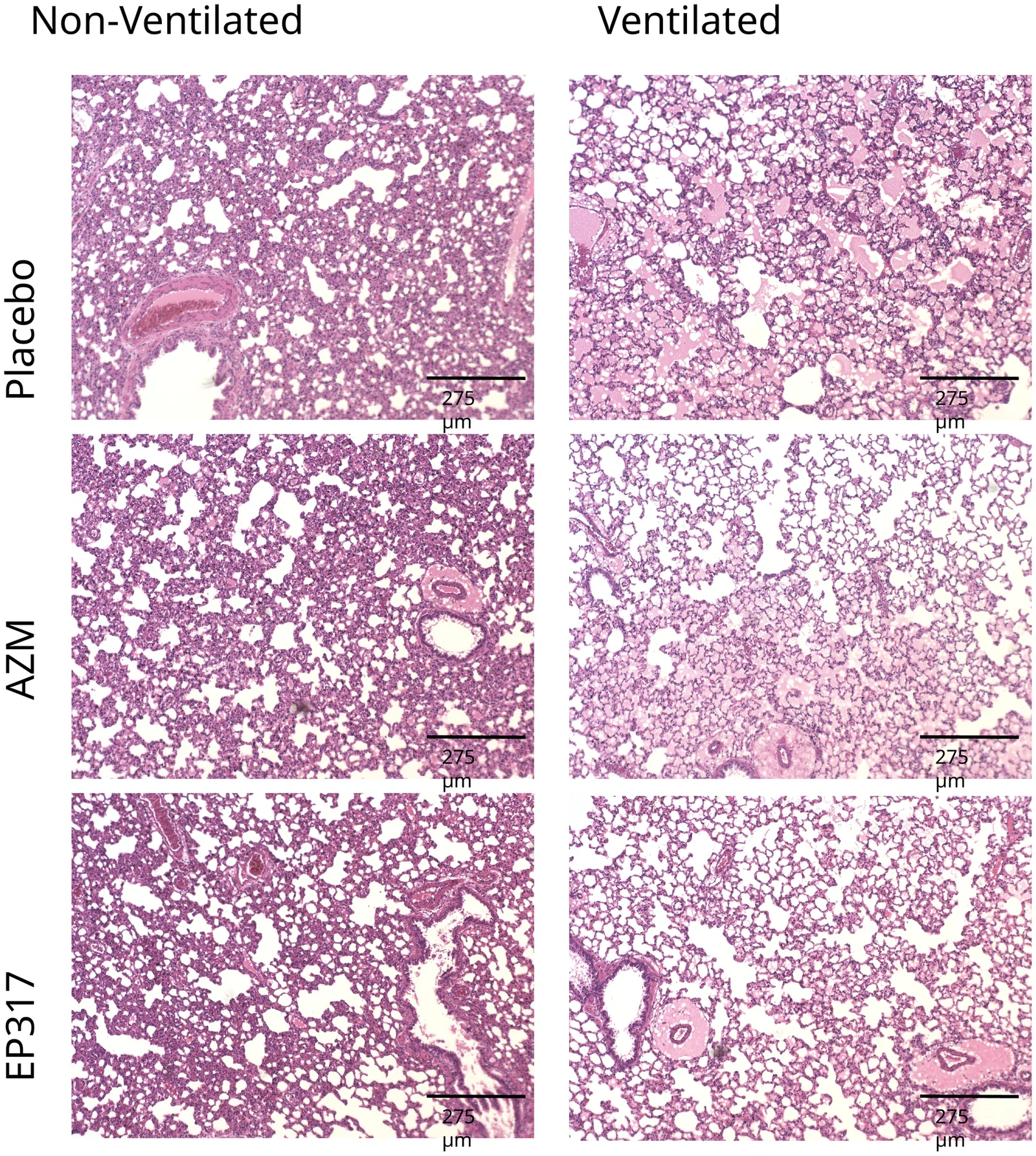
Histopathological assessment reveals reduced alveolar oedema in lungs of rats pre-treated with AZM or EP317. Representative hematoxylin and eosin (H&E)-stained lung tissue sections from non-ventilated controls (PC, AC, EC) and ventilated animals (PV, AV, EV). Placebo-ventilated animals (PV) exhibited marked alveolar oedema (Grade 3) with extensive fluid accumulation in alveolar spaces. AZM-ventilated lungs (AV) showed mild alveolar oedema in most samples. EP317-ventilated animals (EV) demonstrated minimal alveolar oedema and preserved alveolar architecture despite exposure to identical injurious ventilation parameters (PIP 35 cmH_2_O/PEEP 5 cmH_2_O). Non-ventilated control groups (PC, AC, EC) displayed normal lung histology. Isolated foci of perivascular or subpleural mononuclear inflammation observed across groups, including controls, were interpreted as incidental background findings. Scale bar = 275 μm.

Histological examination of H&E-stained lung sections revealed varying degrees of alveolar oedema and inflammatory changes across the experimental groups (Fig 5; whole lung sections are shown in S1 Fig). In the placebo ventilated group (PV), alveolar oedema was clearly present, with microphotographic evidence showing marked oedema (Grade 3). In contrast, lungs from non-ventilated animals in the placebo (PC), AZM-treated (AC), and EP317-treated (EC) groups showed normal histological appearance.

In the AZM-ventilated group (AV), histopathology revealed mild alveolar oedema in most samples. Notably, the EP317-ventilated group (EV) exhibited minimal alveolar oedema and overall less severe histological damage compared with the AV and PV groups. Despite being subjected to the same mechanical ventilation pressure (PIP 35 cmH_2_O/PEEP 5 cmH_2_O), rats in the EV group appeared to preserve better alveolar structure more effectively.

Isolated foci of perivascular or subpleural mononuclear inflammation were observed in individual samples across all groups, including control animals, suggesting these changes may represent incidental findings within the background range for this rat strain. Two cases of osseous metaplasia were reported in one placebo animal and one AZM-ventilated animal. No hyaline membranes, haemorrhages, capillary congestion, or marked inflammatory infiltration were detected.

Note that lung inflation in the non-ventilated placebo (PC) group appeared variable and, in some samples, less complete than in other non-ventilated groups; this most likely reflects technical variability in the post-mortem instillation/fixation procedure rather than a biological difference, as these animals received no experimental intervention that would be expected to affect baseline lung compliance. This is consistent with the blinded histopathological evaluation, which found no morphological abnormalities in the PC group beyond normal background findings.

### Global gene expression profiling highlights differential immunomodulatory effects of AZM and EP317 in ventilated lung tissue

**Fig 6:**
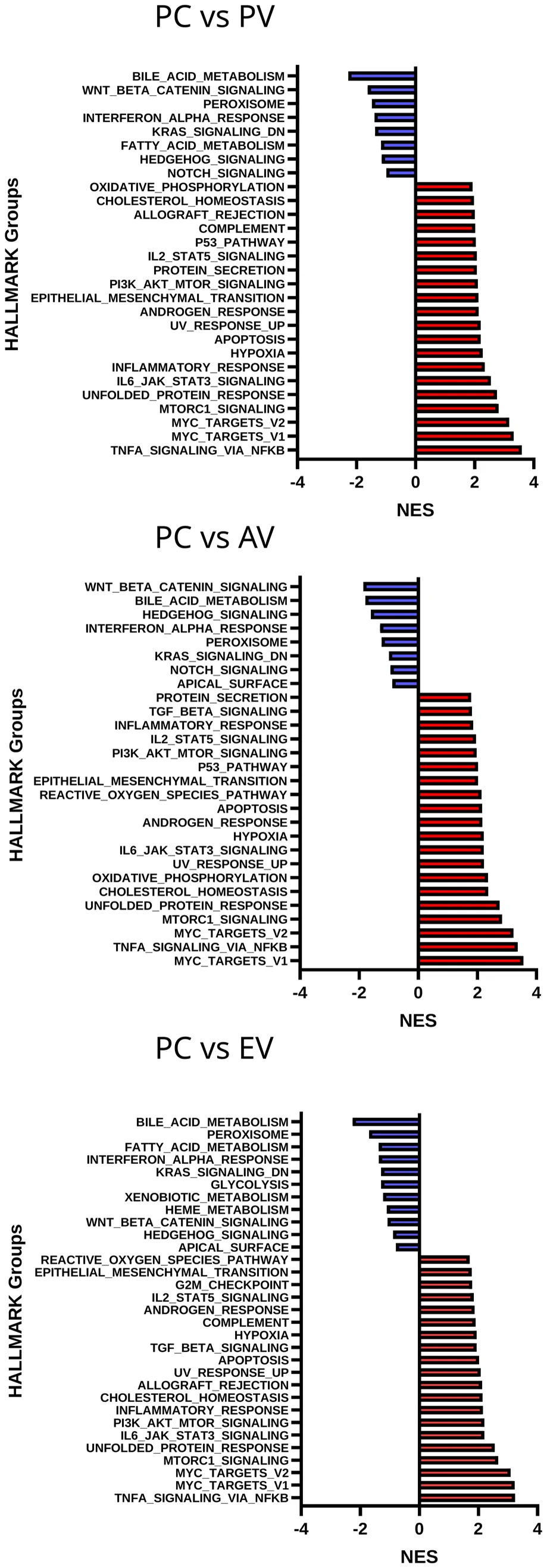
Gene set enrichment analysis reveals differential modulation of inflammatory and stress response pathways by AZM and EP317 in ventilated lung tissue. GSEA comparing global gene expression profiles between non-ventilated placebo controls (PC) and ventilated groups. (Top panel) Comparison of PC versus placebo-ventilated (PV) animals shows robust upregulation of hallmark inflammatory and cellular stress pathways, including TNFα signaling via NFκB (normalized enrichment score, NES = 3.66), IL-6/JAK/STAT3 signaling (NES = 2.57), unfolded protein response, mTORC1 signaling, hypoxia, and apoptosis (all FDR q < 0.001), consistent with acute ventilation-induced lung injury. (Middle and bottom panels) Comparisons of PC versus AZM-ventilated (AV) and PC versus EP317-ventilated (EV) groups demonstrate partial attenuation of inflammatory pathway enrichment following pre-treatment, with modest reductions in NES values. TNFα/NFκB and MYC target gene sets remained significantly enriched in both treatment groups despite pre-treatment. RNA was extracted from whole lung lobe homogenates, and RNA sequencing was performed on n = 4 biological replicates per group. GSEA was conducted using the Hallmark gene set collection from the Molecular Signatures Database (MSigDB). Normalized enrichment scores and false discovery rates are indicated for each pathway.

Gene set enrichment analysis (GSEA) comparing ventilated and non-ventilated placebo groups (PC vs PV) revealed a robust upregulation of hallmark inflammatory and stress response pathways in the lung tissue of ventilated animals (Fig 6). These included TNFα signalling via NFκB (Normalized enrichment score, NES = 3.66), IL-6/JAK/STAT3 signalling (NES = 2.57), unfolded protein response, mTORC1 signalling, hypoxia, and apoptosis. All pathways showed strong statistical significance (False discovery rate, FDR q < 0.001).

This profile is consistent with the acute inflammatory injury characteristic of VILI. AZM and EP317 pre-treatment (PC vs AV and PC vs EV) led to partial attenuation of this response, reflected by modest reductions in NES across several key pathways. Although inflammation-associated gene sets remained significantly upregulated in ventilated samples, notably, TNFα/NFκB and MYC target gene sets continued to show strong enrichment.

## Discussion

The present study evaluated the effects of mechanical ventilation on inflammation and lung injury, as well as the protective properties of AZM and its non-antimicrobial derivative EP317 in a rat model of VILI. In this model, Sprague-Dawley rats were mechanically ventilated at 35 cmH_2_O and 60 breaths per minute for 2 hours. Pre-treatment with AZM or EP317 for 7 days markedly attenuated lung injury compared with placebo, as demonstrated by reduced pulmonary oedema, lower concentrations of circulating inflammatory markers, and improved survival.

Survival outcomes revealed a striking protective effect of both compounds during the 2-hour ventilation protocol. In the placebo-ventilated group, six of eight rats succumbed to VILI within 90 minutes or shortly thereafter. In contrast, seven of eight AZM-pretreated rats survived the full 2-hour ventilation period, with only one animal failing to complete the protocol. Notably, all eight EP317-pretreated rats survived the full 2-hour ventilation period without premature termination.

Analysis of total protein concentrations in BALF revealed a clear trend toward increased protein levels in ventilated placebo rats compared to those pre-treated with AZM or EP317, although this difference did not reach statistical significance. This suggests that mechanical ventilation at high airway pressures consistently promotes disruption of the alveolar-capillary barrier leading to protein leakage, whereas the modest attenuation seen with AZM and EP317 pre-treatment indicates only limited protective effects on barrier integrity. Notably, baseline protein concentrations in non-ventilated, drug-pretreated animals (AC and EC groups) were lower than those observed in non-ventilated placebo controls, raising the possibility that both AZM and EP317 may also exert subtle stabilizing effects on basal barrier function independent of ventilation-induced injury. An effect which could be beneficial in other respiratory diseases [14]. Overall, these results underscore the persistent nature of barrier dysfunction during VILI and highlight a potential benefit conferred by macrolide drug pre-treatment on basal alveolar-capillary integrity.

Wet/dry ratio reflects net lung water content, which is determined by the balance between fluid entering the alveolar and interstitial space (via increased vascular and epithelial permeability) and fluid actively cleared from these spaces (principally via alveolar epithelial Na + /K + -ATPase-driven sodium and fluid transport) [15]. BALF total protein, in contrast, is a relatively direct readout of macromolecular (paracellular) permeability across the alveolar-capillary barrier, independent of clearance capacity. Macrolides, including azithromycin, have been reported to support epithelial barrier and ion transport function in airway and alveolar epithelial models [2], raising the possibility that AZM and EP317 pre-treatment preserve active fluid clearance capacity – producing the modest but significant reduction in wet/dry ratio we observed – without necessarily closing the paracellular leak pathway responsible for protein extravasation, which would explain the absence of a significant treatment effect on BALF total protein. We present this as a plausible mechanistic hypothesis rather than a proven mechanism, and it is an important direction for future work using direct measures of alveolar fluid clearance (e.g., radiolabelled albumin clearance assays).

Mechanical ventilation provoked a robust inflammatory response both locally within the lungs and systematically. Cytokine levels in BALF and blood were significantly elevated after at least 90 minutes of high-pressure ventilation, indicating activation of inflammatory pathways. These findings suggest that injurious ventilation initiates a local pulmonary inflammatory cascade that can extend into the systemic circulation. The local response is likely driven by alveolar epithelial stretch, disruption of the alveolar–capillary barrier, and recruitment and activation of immune cells such as neutrophils and macrophages [16,17]. The corresponding systemic rise in cytokines likely reflects their spillover into the bloodstream, promoting the development of a secondary systemic inflammatory response [18]. Notably, pre-treatment with AZM or EP317 resulted in a less pronounced increase in plasma cytokine levels, while the cytokine expression in the BALF samples showed no such attenuation. These results suggest that AZM and EP317 could modify the pattern of cytokine response triggered by injurious ventilation, primarily affecting systemic response. AZM is well-established as an immunomodulatory agent capable of suppressing pro-inflammatory cytokine production and altering macrophage activation states without inducing overt immunosuppression [19]. AZM modulates monocytes and macrophages, reducing the release of key cytokines including IL-6 and TNF-α largely through interference with NF-κB signalling and related inflammatory pathways [19]. These immunomodulatory properties are well-documented across diverse inflammatory conditions [20]. Our findings align with this established profile: AZM and EP317 pre-treatment likely attenuate the activation of circulating immune cells, including neutrophils, monocytes and T lymphocytes, thereby reducing systemic cytokine output as reflected in the lower plasma cytokine levels. The pronounced elevation of specific cytokines during mechanical ventilation, notably IL-6, MIP-3α (CCL20), GRO-KC (CXCL1), IL-17A, and G-CSF, supports the central role of neutrophil-driven inflammation in VILI pathogenesis. IL-6 is a key mediator associated with lung injury severity, while GRO-KC functions as a potent neutrophil chemoattractant, with their concurrent elevation consistent with robust neutrophil recruitment to injured lung tissue [21,22]. Parallel increases in L-selectin, PAI-1, MCP-3 (CCL7), and RAGE further support this neutrophil-centric inflammatory response, mediating neutrophil adhesion, regulating inflammatory cell influx, enhancing chemotaxis, and amplifying downstream inflammatory signalling, respectively [21,23–26].

The compartmentalized pattern we observed – pronounced attenuation of the systemic (plasma) cytokine response with minimal effect on the local (BALF) cytokine response – may reflect several non-exclusive mechanisms. First, published pharmacokinetic data on azithromycin show that orally administered macrolides achieve comparatively low plasma concentrations but accumulate to very high, sustained concentrations within phagocytic cells, including circulating leukocytes and resident alveolar macrophages, whereas concentrations in the alveolar epithelial lining fluid (ELF), although elevated relative to plasma, remain substantially lower than these intracellular, cell-associated concentrations [27–29]. Because BALF cytokine measurements largely reflect this extracellular, drug-diluted ELF compartment rather than the intracellular leukocyte/macrophage pool in which the drug concentrates, a mismatch between drug exposure and cytokine sampling site could contribute to a plasma-selective suppressive effect, independent of any change in resident alveolar macrophage or epithelial cell drug exposure per se. Second, by the 120-minute endpoint, resident pulmonary immune and epithelial cells may already be committed to a cytokine-secretion program triggered by direct mechanical stretch and barotrauma [30,31], a stimulus that is largely independent of the systemic immunomodulatory pathways (e.g., NF-κB and ERK1/2 inhibition in circulating monocytes) through which macrolides are thought to act [32]; in contrast, the systemic cytokine elevation is thought to arise substantially from spillover of locally produced mediators into the circulation combined with activation of circulating leukocytes, and macrolide-mediated suppression of the latter compartment could account for the plasma-selective effect even without altering local BALF cytokine output. Finally, our two-way ANOVA identified significant treatment x ventilation interaction terms for several plasma cytokines (PAI-1, MCP-3, RAGE, MIP-3α, IL-10, RANTES) but not for the corresponding BALF analytes (with the exception of GRO-KC), providing statistical, not just descriptive, support for a genuine and compartment-specific drug effect rather than a general trend.

One likely contributor to early mortality in VILI is the development of acute pulmonary oedema and its associated consequences [33]. In placebo-treated rats, mechanical ventilation induced pulmonary oedema, as evidenced by a significant increase in the lung wet-to-dry (W/D) weight ratio. Histological analysis confirmed this finding, revealing alveolar oedema and clear structural injury.

Despite the high mortality and pronounced local and systemic inflammatory responses in the placebo-ventilated group, histological injury at the study endpoint was graded as no worse than moderate (Grade 3) alveolar oedema, without hyaline membrane formation, haemorrhage, or dense inflammatory infiltration. We propose several explanations for this apparent discordance. Death in this model likely results predominantly from acute mechanical and haemodynamic derangement – barotrauma-associated impairment of venous return and right heart strain, and severe hypoxaemia – rather than from irreversible structural destruction of lung parenchyma within the brief two-hour experimental window; histological injury typically evolves over a longer timescale than the acute physiological compromise that determines short-term survival. Further, formalin-fixed, paraffin-embedded sections capture a single time point and may under-represent transient interstitial and alveolar fluid that redistributes or is lost during tissue processing, whereas the wet/dry ratio and BALF protein measurements, obtained immediately at sacrifice, capture this fluid more directly.

Another explanation of early mortality might be the cytokine storm caused by VILI, inducing development of multiorgan failure, including cardiac dysfunction [34], which AZM and EP317 were able to attenuate showing lower levels of cytokines in systemic circulation compared to placebo.

Global gene expression analysis by RNA sequencing revealed profound molecular alterations in response to mechanical ventilation. Placebo-treated ventilated rats exhibited substantial changes in pulmonary gene expression, with widespread activation and suppression of genes and pathways in response to mechanical stress. GSEA revealed a marked enrichment of key inflammatory and cellular stress pathways, including TNFα signalling via NF-κB and IL-6/JAK/STAT3 signalling, as well as pathways related to the unfolded protein response, mTORC1 activation, hypoxia, and apoptosis, each demonstrating strong statistical support. These findings highlight that VILI constitutes a complex molecular syndrome extending beyond structural injury, encompassing upregulation of inflammatory genes, activation of stress-response pathways, and engagement of injury-repair signals triggered by alveolar over-distension. Pre-treatment with AZM or EP317 produced modest attenuation of the transcriptional response, with small reductions in normalized enrichment scores across key pathways. While the protective effect was evident, inflammation-associated gene sets remained substantially elevated in both treated groups, indicating that pharmacological intervention only partially modulates the gene regulatory networks activated by mechanical ventilation [35]. AZM is known for its time-dependent actions [36], thus it would be of interest to monitor both the gene expression and cytokine levels at several time points post VILI, to evaluate the treatments for accelerated recovery.

The model used in this study is of clinical relevance. The PIP of 35 cmH_2_O was intentionally chosen to ensure consistent and reproducible lung injury within the 2-hour experimental window, and was decided according to our systematic review of rat VILI models [11]. In clinical practice, a lung protective strategy is typically employed, targeting tidal volumes of 6 mL/kg and plateau pressures below 30 cmH_2_O. However, in diseased lungs, VILI can occur at those pressure levels. Nevertheless, the translational relevance of findings obtained at these pressures should of course be interpreted with appropriate caution.

A limitation of this study is that the sample size (n = 8 per group, with n = 4 per group for each of the BALF/plasma and lung-tissue sub-cohorts) was not derived from a formal a priori power calculation, but from precedent in the published VILI literature; as a result, some secondary endpoints with smaller effective sample sizes after quality-control exclusion of individual assay values may have been underpowered to detect modest treatment effects, particularly for the two-way ANOVA interaction term, which requires larger samples than the main effects to achieve adequate power.

Another limitation of this study is its pre-treatment design, in which AZM or EP317 were administered for seven days prior to the induction of lung injury, rather than after ventilation had already begun. While this design provided a controlled, mechanistically clean test of whether these compounds can favourably precondition the lung and systemic immune system against a subsequent injurious stimulus, it does not directly model the more common clinical scenario in which a patient requiring mechanical ventilation would receive pharmacological intervention only after ventilation, and often injury, has already commenced. The pretreatment paradigm is nonetheless relevant to specific clinical contexts, such as patients with a known, anticipated need for prolonged mechanical ventilation (e.g., planned high-risk cardiothoracic surgery) or patients already receiving azithromycin for an unrelated indication (e.g., chronic macrolide therapy for COPD or bronchiectasis) at the time ventilation becomes necessary. Establishing whether AZM or EP317 retain efficacy when administered as rescue therapy after the onset of ventilator-induced lung injury is an important next step and is a primary focus of our ongoing follow-up studies.

Given the financial relationships between several authors and EpiEndo Pharmaceuticals (the developer of EP317), several measures were taken to safeguard the objectivity of this study. Histopathological assessment, the most subjective outcome measure, was performed by an independent, blinded commercial pathology laboratory (PatConsult) with no financial relationship to EpiEndo. All biochemical and molecular endpoints (Luminex multiplex cytokine assays, BCA protein assay, RNA sequencing and differential expression analysis) were generated using standardized, instrument-read protocols with pre-defined analysis pipelines, minimizing scope for subjective bias in data generation. Animal husbandry, dosing, ventilation, and sample collection were performed by academic co-authors at the University of Iceland and Landspitali-University Hospital, institutions independent of EpiEndo.

In conclusion, this study demonstrates that VILI arises from a complex interplay of mechanical trauma and inflammation, with high-pressure ventilation producing severe barrier disruption, pulmonary oedema, and increased mortality. Remarkably, pre-treatment with AZM or its derivative EP317 conferred significant protective effects, substantially improving survival, reducing the degree of ventilation-induced pulmonary oedema, and attenuating the induction of pro-inflammatory cytokines in plasma. While these compounds did not fully prevent local lung inflammation or structural injury within the pulmonary compartment, their immunomodulatory actions mitigated key systemic consequences of VILI. These findings highlight the need for multifaceted therapeutic strategies that address both mechanical and immunological drivers of lung injury, with AZM and EP317 offering a promising foundation for future intervention.

## Supporting information

S1 Fig Whole lung sections. Whole rat lung sections. H&E-stained lung tissues from non-ventilated controls (PC, AC, EC) and ventilated animals (PV, AV, EV). Box width = 1300 μm.
(TIF)
